# Comparison of 4D Phase-Contrast MRI Flow Measurements to Computational Fluid Dynamics Simulations of Cerebrospinal Fluid Motion in the Cervical Spine

**DOI:** 10.1371/journal.pone.0052284

**Published:** 2012-12-21

**Authors:** Theresia I. Yiallourou, Jan Robert Kröger, Nikolaos Stergiopulos, David Maintz, Bryn A. Martin, Alexander C. Bunck

**Affiliations:** 1 Laboratory of Hemodynamics and Cardiovascular Technology, École Polytechnique Fédérale de Lausanne, Lausanne, Switzerland; 2 Department of Clinical Radiology, University Hospital of Münster, Münster, Germany; 3 Department of Radiology, University Hospital of Cologne, Cologne, Germany; 4 Conquer Chiari Research Center, University of Akron, Akron, Ohio, United States of America; Tokyo Metropolitan Institute of Medical Science, Japan

## Abstract

Cerebrospinal fluid (CSF) dynamics in the cervical spinal subarachnoid space (SSS) have been thought to be important to help diagnose and assess craniospinal disorders such as Chiari I malformation (CM). In this study we obtained time-resolved three directional velocity encoded phase-contrast MRI (4D PC MRI) in three healthy volunteers and four CM patients and compared the 4D PC MRI measurements to subject-specific 3D computational fluid dynamics (CFD) simulations. The CFD simulations considered the geometry to be rigid-walled and did not include small anatomical structures such as nerve roots, denticulate ligaments and arachnoid trabeculae. Results were compared at nine axial planes along the cervical SSS in terms of peak CSF velocities in both the cranial and caudal direction and visual interpretation of thru-plane velocity profiles. 4D PC MRI peak CSF velocities were consistently greater than the CFD peak velocities and these differences were more pronounced in CM patients than in healthy subjects. In the upper cervical SSS of CM patients the 4D PC MRI quantified stronger fluid jets than the CFD. Visual interpretation of the 4D PC MRI thru-plane velocity profiles showed greater pulsatile movement of CSF in the anterior SSS in comparison to the posterior and reduction in local CSF velocities near nerve roots. CFD velocity profiles were relatively uniform around the spinal cord for all subjects. This study represents the first comparison of 4D PC MRI measurements to CFD of CSF flow in the cervical SSS. The results highlight the utility of 4D PC MRI for evaluation of complex CSF dynamics and the need for improvement of CFD methodology. Future studies are needed to investigate whether integration of fine anatomical structures and gross motion of the brain and/or spinal cord into the computational model will lead to a better agreement between the two techniques.

## Introduction

Cerebrospinal fluid (CSF) dynamics have been examined in craniospinal disorders because analysis of brain and spinal cord morphology alone has been insufficient to explain patient symptoms and surgical outcome [1], [2]. Single-slice 2D phase contrast MR flow imaging (2D PC MRI) in the sagittal or axial orientation has been used to quantify CSF hydrodynamic parameters such as peak CSF velocities and jets in Chiari I malformation (CM) [3], [4], relative timing of CSF and arterial pulsations [5], [6], [7] and pulse wave velocity in the spinal subarachnoid space (SSS) [8]. However, the unidirectional encoding of 2D PC MRI CSF flow measurements does not permit quantification of 3D complexities within the CSF flow field [9].

Time-resolved three-directional velocity encoded phase contrast MR imaging (4D PC MRI) has been increasingly appreciated for its potential to quantitatively and qualitatively assess CSF flow dynamics and provide insight into complex flow phenomena such as secondary flow and vortex strength that can occur in craniospinal disorders [1], [10]. Bunck et al. [11] found that 4D PC MRI resulted in detection of greater CSF peak velocities than single-plane 2D PC MRI measurements when assessing CSF flow in CM patients with and without a syrinx. 4D PC MRI has also been utilized to investigate the CSF flow field in the ventricles of the brain [12] and in hydrocephalus patients [13].

**Figure 1:**
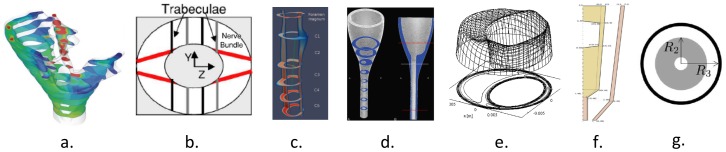
Varying levels of anatomical assumptions in the literature when simulating CSF in the cervical spine. (Decreasing level of anatomical complexity from left to right, respectively). a) A subject-specific rigid wall geometry with CSF moving within a SSS of anisotropic porosity [15]. b) An idealized 2D SSS geometry including spinal cord nerve roots, arachnoid trabeculae and denticulate ligaments in a symmetric arrangement around the spinal cord [20]. c) A subject-specific 3D SSS geometry without small anatomical structures and geometric smoothing [23]. d) An idealized 3D geometry of a healthy subject [22]. e) The first simulation of CSF in the cervical SSS idealized as two concentric ellipses [14]. f) A 2D axisymmetric spinal cord and dura model with moving walls [30]. g) A 2D axisymmetric model of wave propagation in the spine based on an analytical solution of concentric elastic tubes [28]. Refer to Table 1 for details in each simulation.

To date, the CSF flow field obtained by 4D PC MRI has not been compared to 3D computational fluid dynamics (CFD) simulations; a helpful tool to quantify the CSF movement within the SSS and intracranial space [14], [15], [16], [17], [18], [19]. CFD simulations are uniquely suited for variational analysis; a technique that can be used to help assess the importance of individual anatomical aspects of the CSF system such as the spinal cord nerve roots or tonsillar descent in CM. Figure 1 summarizes the existing computational simulation studies of the cervical SSS CSF motion under varying levels of complexity. In accordance with Figure 1, Table 1 provides details for the computational studies and their anatomical simplifications. Loth et al. [14] conducted the first rigid wall CFD simulation of the CSF movement in the SSS. Small anatomical structures such as the spinal cord (SC) nerve roots, denticulate ligaments and arachnoid trabeculae were not included in the simulated geometry. Stockman [20] investigated the impact of small anatomical structures on the CSF flow field and found that the velocity profiles were not significantly affected by the presence of the fine structures when the spacing was symmetric around the SC. Subsequent to these studies it has generally been assumed that small structures in the SSS do not have a significant impact on macro-scale CSF velocity profiles.

**Table 1:** Literature review of computational simulations of CSF motion in the cervical SSS and/or craniospinal junction in healthy conditions and patients with craniospinal disorders.

Author	Technique	Geometry	Tissue motion	Arachnoid trabeculae	Nerve roots	
Gupta et al. [15]	CFD, anisotropic porous media	3D subject-specific	No	Yes	No	
Stockman et al. [20]	CFD, Lattice Boltzmann	2D idealized	No	Yes	Yes	
Roldan et al. [21]	3D rigid wall CFD	3D subject-specific	No	No	No	
Linge et al. [22]	3D rigid wall CFD	3D idealized	No	No	No	
Loth et al. [14]	3D rigid wall CFD	2D concentric ellipse based on subject	No	No	No	
Rutkowska et al. [23]	3D rigid wall CFD	3D patient specific	No	No	No	
Bertram [30]	Numerical model/wave propagation	2D idealized axisymmetric, tapered tubes	Yes	No	No	
Cirovic [28]	Numerical model/wave propagation	2D concentric tube with constant diameter	Yes	No	No	
Carpenter et al. [48],Elliott et al. [49],Cirovic et al. [50]	Numerical model/wave propagation	1D coaxial, fluid-filled, elastic tubes	Yes	No	No	
Elliott et al. [51]	Two multiple-compartment hydraulic circuit models	1D coaxial, fluid-filled, permeable tubes	No	No	No	
Linninger et al. [52]	FSI	Multi compartment model of intracranial dynamics	Yes	No	No	
Bilston et al. [53]	CFD	2D Axisymmetric, cylindrical model	Yes	No	No	

Abbreviations: CSF = cerebrospinal fluid, SSS = spinal subarachnoid space, CFD = computational fluid dynamics, FSI = fluid-structure interaction.

Roldan et al. [21] simulated CSF in rigid geometrically realistic SSS models without fine structures based on MRI measurements. The results indicated heterogeneous CSF flow fields with anterolateral flow jets around the SC [2]. Linge et al. [22] examined the effect of anatomic variation on CSF dynamics without fine anatomy and found the spatial variations in flow patterns to resemble those observed in PC MR studies. Rutkowska et al. [23] compared 3D rigid wall subject-specific CFD simulations of cyclic CSF flow to 2D PC MR measurements in CM patients, patients who had previous craniovertebral decompression and controls and observed that the various CSF flow patterns were greater in Chiari patients than in controls. In contrast to other studies assuming the subarachnoid space to be a strictly fluid space, Gupta et al. [15], [16] conducted a study to simulate CSF movement within a uniformly distributed anisotropic porous media representative of the arachnoid trabeculae. Their results supported that the arachnoid trabeculae density and dimensions had a significant impact on pressure gradients and would alter kinetics of drug distribution within the CSF system.

Several authors have simulated the CSF flow field and spinal tissue displacement considering the SSS to be an axisymmetric coaxial elastic tube system [24], [25], [26], [27], [28], [29], [30]. These simulations are based on analytical solutions for wave propagation within tubes or 2D axisymmetric fluid-structure interaction simulations with simplified boundary conditions from *in vivo*. These models helped to further understand the impact a stenosis and/or syrinx can have on wave propagation in the SSS and the internal stresses that might arise within the neural tissue. Martin et al. [31], [32], [33] conducted *in vitro* experiments to examine the importance of spinal stenosis and presence of a non-communicating syrinx on spinal CSF dynamics. Bottan et al. [34] constructed a 3D phantom model of the intracranial pressure and CSF dynamics. As a whole, the *in vitro* experiments and axisymmetric models, despite many anatomical simplifications, emphasized the importance of mechanical properties of the neural tissue such as compliance and permeability and the complex fluid-structure interaction involved with the CSF flow obstruction and neural tissue.

Altogether these different approaches aiming to simulate CSF dynamics warrant verification by *in vivo* measurements in order to assess the extent to which the different models reflect *in vivo*. At present, 4D PC MRI can be regarded as the method that offers the best and most comprehensive insight into *in vivo* CSF dynamics. For that reason it is most suitable for a comparison to CFD models.

The aim of the present study was to compare the CSF flow field in the cervical spine, measured by a) 4D PC MRI flow imaging and b) simulated by subject specific CFD, under a variety of CSF flow conditions (age and pathology). A variety of CSF flow conditions were examined by choosing a heterogeneous subject group of healthy volunteers and CM patients at different ages. For each subject we compared the 4D PC MRI to the CFD flow field in terms of 1) peak velocities and 2) velocity profiles. Our hypothesis was that important differences would be present between the CFD simulations and the 4D PC MRI measurements due to neglect of the small structures and tissue motion in the CFD simulations.

## Materials and Methods

### Ethics Statement

The MR data acquisition was performed at the Department of Radiology of Münster. The study was approved by the institutional review board of the University of Münster. Before the MR exams, written informed consent was obtained from all the healthy volunteers and CM patients. Prior to further data processing MR data were anonymized.

### In vivo 4D PC MR Measurements

4D PC MRI CSF velocity measurements were acquired in the cervical spine (from the foramen magnum (FM) to C7 vertebrae level) of three healthy volunteers (Healthy volunteers a, b and c) (aged 24±5 years) with no history of neurological disorder or spinal trauma and four CM patients (CM 1, 2, 3 and 4) (aged 5±2.8 years) (see Table 2 for the summary of the study population). Note that age and sex matching of the healthy volunteers and patients was not sought in this study because the primary focus was to obtain a variety of CSF flow conditions and compare them to subject specific CFD simulations. In addition, neck angulation of the subjects was not controlled.

**Table 2:** Demographic and clinical characteristics of the study population.

Study population	Age/Sex	Disorders	Symptoms	Flow abnormalities	Tonsillar herniation (mm)
Healthy volunteers a-c	28/F, 22/M, 22/M	None	None	None	N/A
CM 1	7/F	CM	Asymptomatic	Unilateral flow jet	28.9
CM 2	7/F	CM	Migraine	Inhomogeneous flow	16.4
CM 3	1/M	CM	Complex syndrome	Bilateral flow jets and bidirectional flow	10.3
CM 4	5/M	CM	Impaired balance, lackof concentration	Unilateral flow jets	5.8

Abbreviations: F = female, M = male, CM = Chiari I malformation.

4D PC MRI measurements were taken on a 1.5 T MRI scanner (Achieva 2.6 scanner, Philips, Best the Netherlands) with a standard 16-channel head and neck coil, using the sequence parameters as described in the protocol by Bunck et al. [1]. In brief, for 4D PC MRI imaging a retrospectively ECG-triggered, T1-weighted, segmented gradient echo sequence (T1-TFE) with a three directional velocity encoding and an isotropic resolution of 1.5 mm was used (reconstructed voxel resolution: 1 mm). Encoding velocity was set to 10 cm/s in healthy volunteers and 20 cm/s in all patients. For PC measurements a local phase correction (LPC) filter provided by the manufacturer was used to subtract the background offset caused by eddy currents. The image volume was aligned in the sagittal plane with the 3D stack covering the craniocervical junction and the entire cervical thecal sac. Imaging time varied between 8 and 14 minutes depending on the individual heart rate and encoding velocity factor.

To define the cervical spine geometry for the CFD simulations, a high resolution T2-weighted 3D, turbo spin-echo sequence (VISTA) with an isotropic spatial resolution of 0.8 mm was obtained. The 3D field of view was adjusted to anatomical dimensions, laterally securely extending beyond the inner confinement of the FM.

Motion of the cerebellar tonsils in the sagittal plane during the cardiac cycle was obtained using a retrospective ECG-triggered balanced TFE sequence with an acquired spatial resolution of 1×1 mm (reconstructed in-plane voxel resolution: 0.4 mm) and a slice thickness of 6 mm. A single slice in the sagittal midplane was acquired with 30 heart phases, 70% phase percentage and a 50° flip angle.

### CFD Simulation

**Figure 2:**
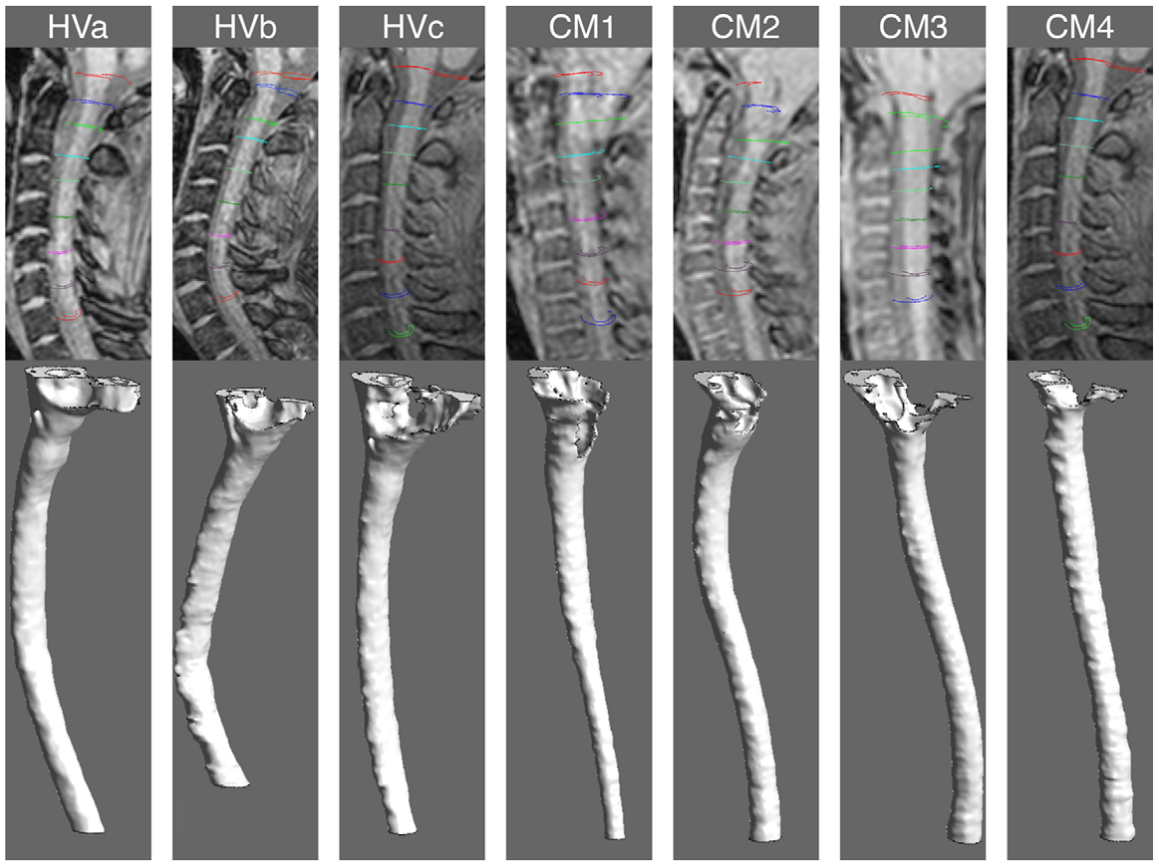
3D reconstruction of the cervical SSS based on manual segmentation. Segmentation of the healthy subjects (left) and CM patients (right). The 3D reconstruction depicts the SSS where the CSF pulsates (between the dura and spinal cord tissue). Note the SSS constriction near the FM in the four CM patients in comparison to the healthy subjects.

The three-dimensional anatomy of the cervical SSS was reconstructed for each subject from the T2 weighted VISTA MRI images with manual segmentation using ITK Snap software (Version 2.2.0, PA) (Figure 2). The lower cervical spine was manually segmented approximately 5 cm caudal to C7, beyond the region of flow comparison. Spinal cord nerve roots, denticulate ligaments and other fine anatomical structures were not taken into account in the segmentation. Careful attention was given to exclude the epidural space outside of the dural confinement. The 3D geometry was smoothed with a Laplacian smoothing using MeshLab software (Version 1.3.0, Italy, Rome). A rigid wall unstructured computational grid was generated within the ANSYS ICEM CFD software (Version 13.0, Canonsburg, PA) consisting of approximately two million tetrahedral elements.

**Figure 3:**
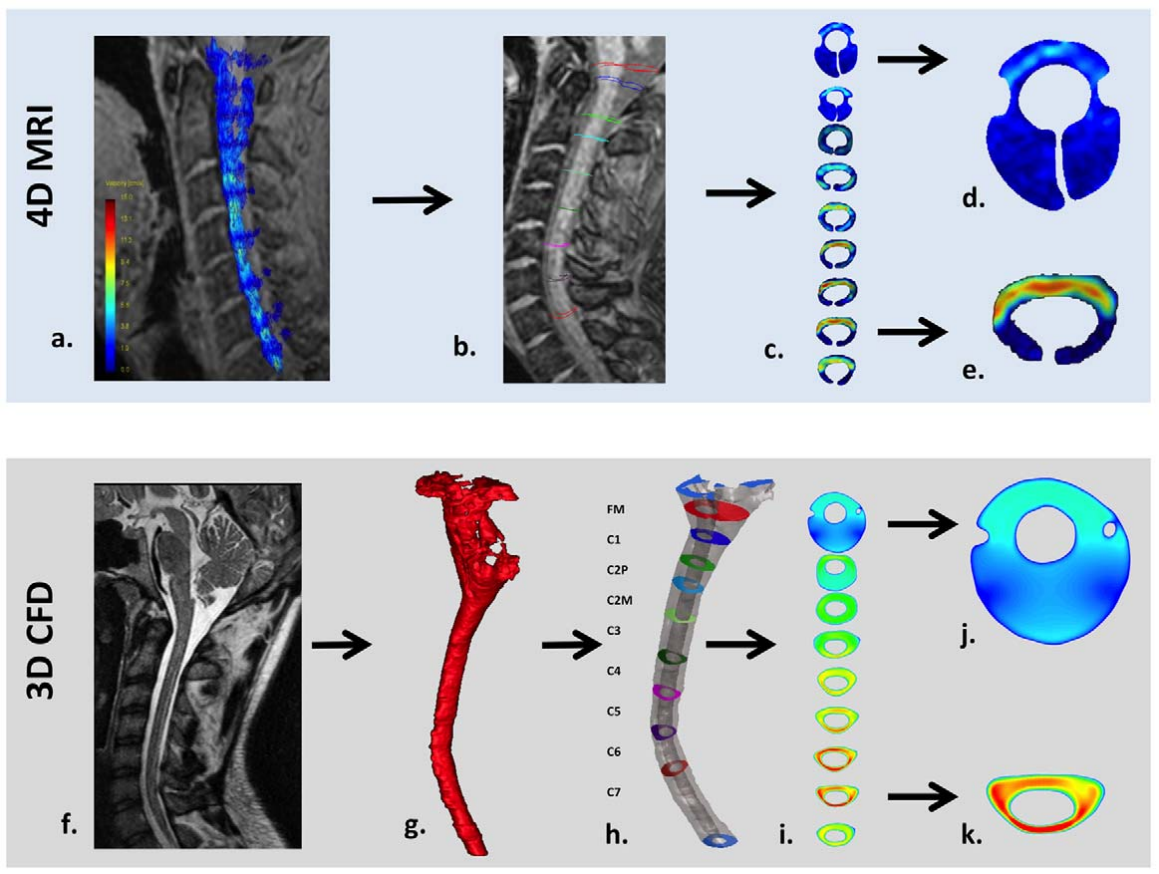
Workflow for 4D PC MRI (top row) and CFD (bottom) methodology in a healthy volunteer. a) 4D PC MRI velocity vectors superimposed on the coarse 2D anatomy scan. b) Placement of axial planes along the cervical SSS and c) 2D velocity profile visualization of the axial planes. d) Velocity profile example at the FM where ROI image truncation was required due to low velocities and noise in the MRI signal (see Methods for details). e) Velocity profile in the lower cervical SSS where the ROI required less image truncation. f) High resolution anatomical MRI scan used to define the geometry for the CFD simulation. g) 3D rendering of the cervical SSS segmentation before end truncation and geometric smoothing. h) 3D rendering of the smoothed cervical SSS geometry and axial planes where the CFD velocity profiles were observed. i) 2D velocity profile plots for each axial location. j) Velocity profile at the FM showing a larger cross-section than the FM in the 4D PC MRI (compare to d). k) Velocity profile in the lower cervical SSS that compares more favorably in terms of ROI size and shape to that observed in the 4D PC MRI (e).

A subject specific CSF flow waveform was imposed for each CFD simulation based on the following methodology (See Figure 3). The CSF flow waveform was obtained at nine axial locations along the SSS (FM to C7) based on the 4D PC MRI measurements for each subject. CSF flow was determined by integrating the pixel velocities within the region of interest (ROI) at each axial location (see data processing and analysis for details on ROI selection). Based on a CFD study by Loth et al. [14], the CSF flow waveform at each axial location was offset so the net CSF flow per cycle was zero (net flow in the SSS is known to be nearly zero). The average offset for all subjects was relatively small compared to the peak flow rates (−0.23±0.10 cm/s).

The CSF flow waveform from the axial location with the greatest peak flow rate was selected for the inlet boundary condition of the CFD simulation. This location was selected to assure that the CFD results did not under predict CSF velocities and because a higher fidelity MRI signal is expected within a ROI with greater CSF movement. For our study, the axial location with the greatest CSF flow rate was located at the C1, C2M or the C3 level for all subjects. Systolic CSF flow occurred in the cranial-caudal direction. Based on the CSF flow waveform, a blunt CSF velocity profile was imposed at the flow inlet on the caudal end of the CFD model (approximately 5 cm below C7). The caudal end of the model was chosen as an inlet to allow for a fully developed velocity profile within the ROI (FM to C7). A no-slip boundary condition was specified at the walls. Similar to other CSF CFD studies in the literature [21] [35], a zero pressure boundary condition was imposed at the flow outlet on the cranial end of each CFD simulation.

The Navier-Stokes equations were solved numerically by the commercial finite volume CFD solver ANSY CFX (Version 13.0, Canonsburg, PA), resulting in a flow velocity vector and a pressure scalar at each point of the computational mesh. CSF was modeled as an incompressible Newtonian fluid with the hydrodynamic characteristics of water at body temperature [36], [37] (density of ρ = 1000 kg/and dynamic viscosity of μ = 0.001Pa*s). Flow was assumed to be laminar. ANSYS CFX uses an element-based finite volume method to solve the Navier-Stokes equations by implementing the Gauss’ Divergence Theorem to convert volume integrals involving divergence and gradient operators to surface integrals. Within the CFX solver settings, the utilized advection scheme had second order accuracy. The utilized transient time-stepping scheme was second order implicit backward Euler. The root mean square residual (RMS) was set to 1*10^−4^ as a convergence criterion. Each CFD simulation took approximately 8 hours to complete in parallel on a computer with 8 processors and 12-GB RAM. The total simulation time was sufficient for temporal periodicity to be established.

Grid and time step independence studies were carried out with the following methodology. Three grid sizes with tetrahedral elements were analyzed having 1,310,000 (coarse), 2,860,000 (medium) and 3,800,000 (fine) elements. Pressure and velocity contours at several cross-sections of the domain were compared at different simulation times during the third simulation flow cycle. We assessed maximum relative error, *e*, based on the following formula,
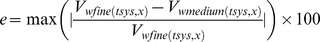
where *V_w_* is the velocity in the z direction calculated at the time step, *tsys*, corresponding to peak systolic flow within the cardiac cycle and *x* is the spatial position along a vector located within each cross-section (axial planes FM, C3 and C7). The subscripts “fine” and “medium” refer to calculations carried out with the fine and medium grid respectively. We used the same formula to estimate the relative error between the coarse and medium grids. Following confirmation that the medium grid was sufficient to capture the important flow features, the CFD simulations were carried out with the medium grid. Time-step independence was assessed by carrying out the computations for the first period using time step sizes of *T*/100, *T*/1,000 and *T*/10,000 where *T* is the length of one cardiac cycle for each subject. The time step size utilized for our presented simulation results was *T*/1000.

### Data Processing and Analysis

**Figure 4:**
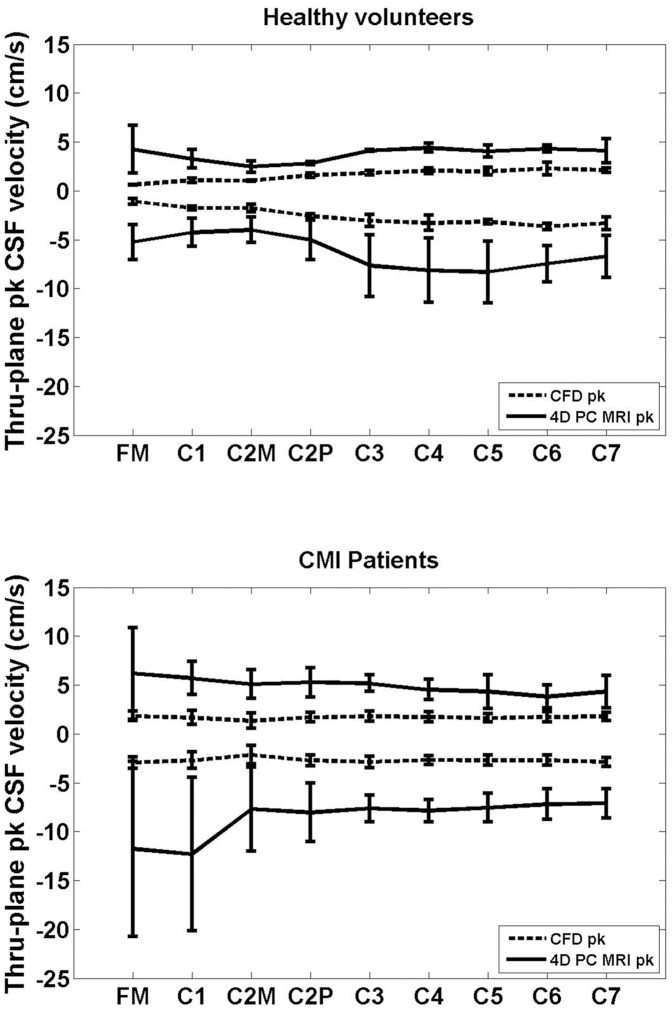
Comparison of the mean thru-plane peak CSF velocities between 4D PC MRI and CFD. Peak systolic and diastolic velocities were measured by the 4D PC MRI and simulated by CFD in the cervical spine (FM-C7, FM is near the head and C7 is towards the feet) in healthy volunteers (Healthy a, b and c) and CM patients (CM 1, 2, 3, and 4). Values are given as mean ± SD (cm/s) for the three healthy subjects (top) and four CM patients (bottom). Positive (diastolic) and negative (systolic) velocities reflect head and foot directed flow, respectively.

Data processing of the 4D PC MRI data sets, flow quantification and flow visualisation was carried out using the GTFlow software (Version 1.6.4, Gyrotools Ltd., Zurich, Switzerland). For flow quantification, the ROIs were manually defined in the axial orientation orthogonal to the spinal axis at the level of the FM and every cervical vertebra including the middle of C2 (FM, C1, C2M, C2P, C3, C4, C5, C6, C7; see Figure 3b for typical ROI orientation and Figure 4). Special care was taken to avoid regions within the ROIs with high velocities that occurred due to vascular blood flow. Differentiation between high CSF flow velocities due to anatomical restrictions and low vascular flow velocities was visually performed based on the PC images by assessing direction of flow over time. While the direction of blood flow does not change over time, i.e. flow is either directed caudally for venous blood or cranially for arterial blood, flow direction of CSF changes from the caudal direction during systole to the cranial direction during diastole. The ROI axial planes with high velocities due to vascular flow were typically located at the FM level near the left and right vertebral and the basilar artery. In some cases the ROI at the FM and C1 required partial truncation due to lack of signal and/or noise in the 4D PC MRI signal (Figure 3c, d, and e) and because of high arterial blood flow velocities. It should be noted that the post-processing software ROI selection was limited to one closed shape region at each axial level. Thus, each ROI had a “cuff” shape located around the spinal cord, with each tip of the cuff located on the posterior side of the spinal cord where lower CSF velocities were present. In the regions where the spinal cord was completely surrounded by CSF, the tips of the “cuff” shaped ROI were adjusted to touch, resulting in a virtually ring-like shape. Overall, the ROI shapes were adjusted to include all relevant flow components by correcting the shape based on the velocity encoded PC images. By these means it was assured that peak velocities were not missed.

The 4D PC MRI measurements and CFD simulation results were compared in terms of 1) peak velocities and 2) visual inspection of the velocity profiles for each ROI along the spine. Axial planes were placed along the CFD simulated geometries with the same orientation and location as the 4D PC MRI ROIs. For each plane the peak thru-plane systolic (caudal) and diastolic (cranial) flow velocities were quantified. For each axial level along the spine the average and standard deviation of the peak caudal and cranial velocities were determined for the three healthy subjects and four CM patients. In addition, the flow was assessed visually to understand any differences in velocity profiles if present. We focused on a) anterior versus posterior flow differences, b) presence of flow jets and c) flow near the nerve roots. The CSF stroke volume (SV) for each ROI along the spine was determined by integrating the absolute value of the CSF flow waveform and dividing the integrated value by two (total pulsatile volume moving through an ROI).

Motion of the cerebellar tonsils during the cardiac cycle was assessed at the mid-sagittal plane near the FM for one healthy volunteer (Healthy c) and four CM patients using the following methodology. Based on the ECG-triggered balanced TFE cine images, the image with the maximum rostral and caudal displacement of the tonsils was selected by visual estimation of the tonsil position. The individual pixel values in the rostral and caudal displacement image were subtracted from one another to produce a transparent threshold image mask (aqua colour) that was overlaid on the original tonsil position with maximum rostral displacement. Thus, the space in the image without any transparent aqua masking corresponded to regions where the tissue moved during the cardiac cycle and vice versa. This provides a visualization of the level of tissue motion in each subject.

## Results

### Peak Velocities

4D PC MRI data sets were acquired for three healthy subjects and four CM patients. The mean thru-plane peak cranial and caudal velocities measured by 4D PC MRI and simulated by CFD at different axial locations along the cervical spine are presented in Figure 4. All velocities are given as mean ± SD cm/s. Positive and negative velocities reflect head and foot directed flow, respectively.

The 4D PC MRI velocity measurements were consistently greater in magnitude than the CFD simulations. For healthy subjects at the FM, 4D PC MRI average peak caudal and cranial velocities were −5.2±1.8 cm/s and 4.2±2.5 cm/s, respectively. In contrast, average CFD velocities at the FM were −1.1±0.3 cm/s and 0.5±0.0 cm/s, respectively in healthy subjects. The difference between 4D PC MRI and CFD velocities was greater in the CM patients. For CM patients at the FM, 4D PC MRI average peak caudal and cranial velocities were −11.8±9.0 cm/s and 6.2±4.7 cm/s, respectively. For CM patients at the FM, average CFD velocities were −2.9±0.6 cm/s and 1.8±0.5 cm/s, respectively.

While the focus of this paper was not to differentiate healthy from CMI patients in terms of their velocities, a number of differences were observed in the two groups. Overall, the 4D PC MRI measurements had a greater standard deviation of peak velocities than the CFD results for both the healthy and CMI patients. In CMI patients the greatest standard deviation of peak velocities occurred in peak systole at the FM and C1 level for the 4D PC MRI measurements. In the healthy group, we noted that the greatest differences between the average CFD peak velocities and the 4D PC MRI peak velocities occurred in systole (caudal directed flow) at the C3 to C6 level. In contrast, in the CMI patients the greatest differences in average peak velocities occurred at the FM and C1 level (Figure 4)).

### Velocity Profiles

**Figure 5:**
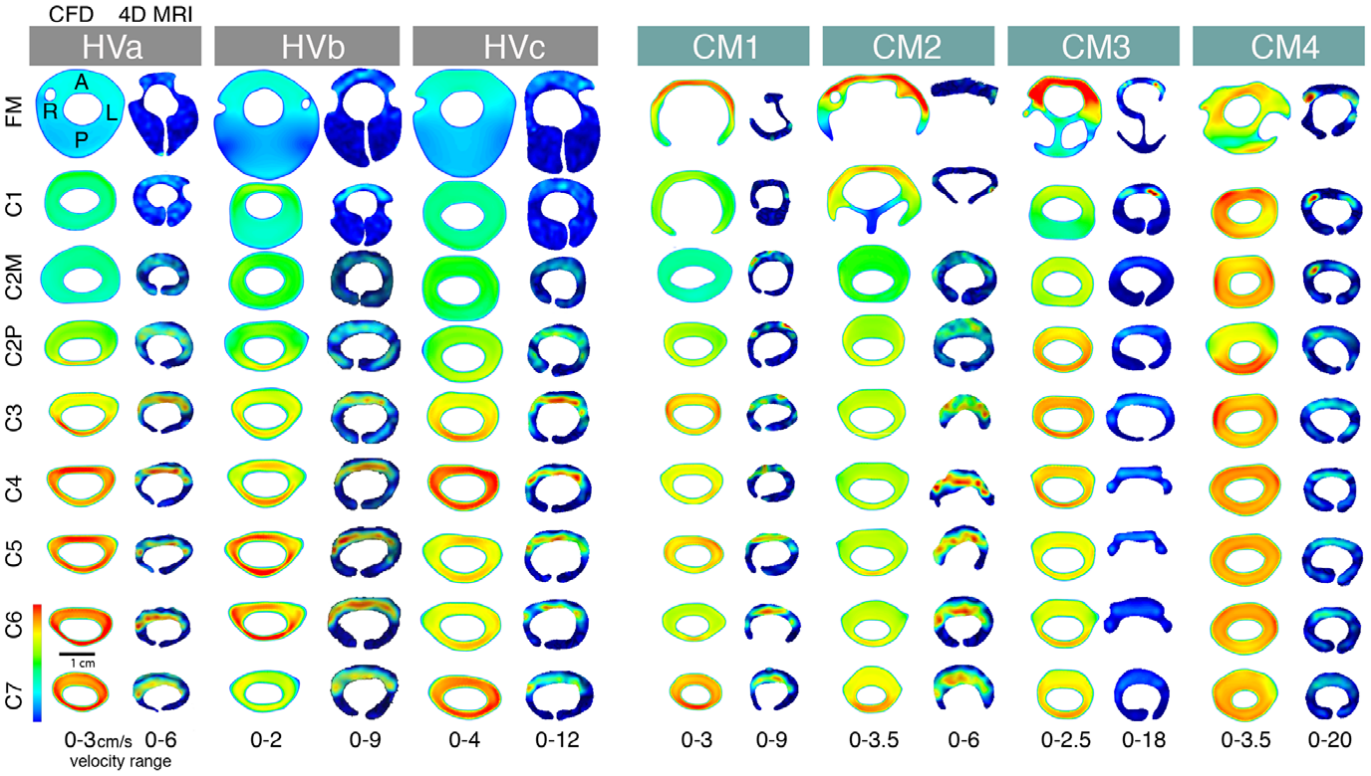
Thru-plane peak CSF velocity profiles (foot direction) at each axial location along the cervical spine. The left and right image for each subject corresponds to the CFD simulation and 4D PC MRI measurements along the cervical spine (FM-C7 level), respectively. CSF velocities were elevated in the anterior SSS in comparison to the posterior space in all of the 4D PC MRI velocity profiles (healthy and patients). The posterior versus anterior flow differences were not present in the CFD results; which maintained a fairly uniform velocity profile around the spinal cord in all simulations except CM 1 and CM 2 near the FM. Note, velocity scales are different for each image (shown at bottom of each image set) so as to highlight the difference in velocity profiles.

**Figure 6:**
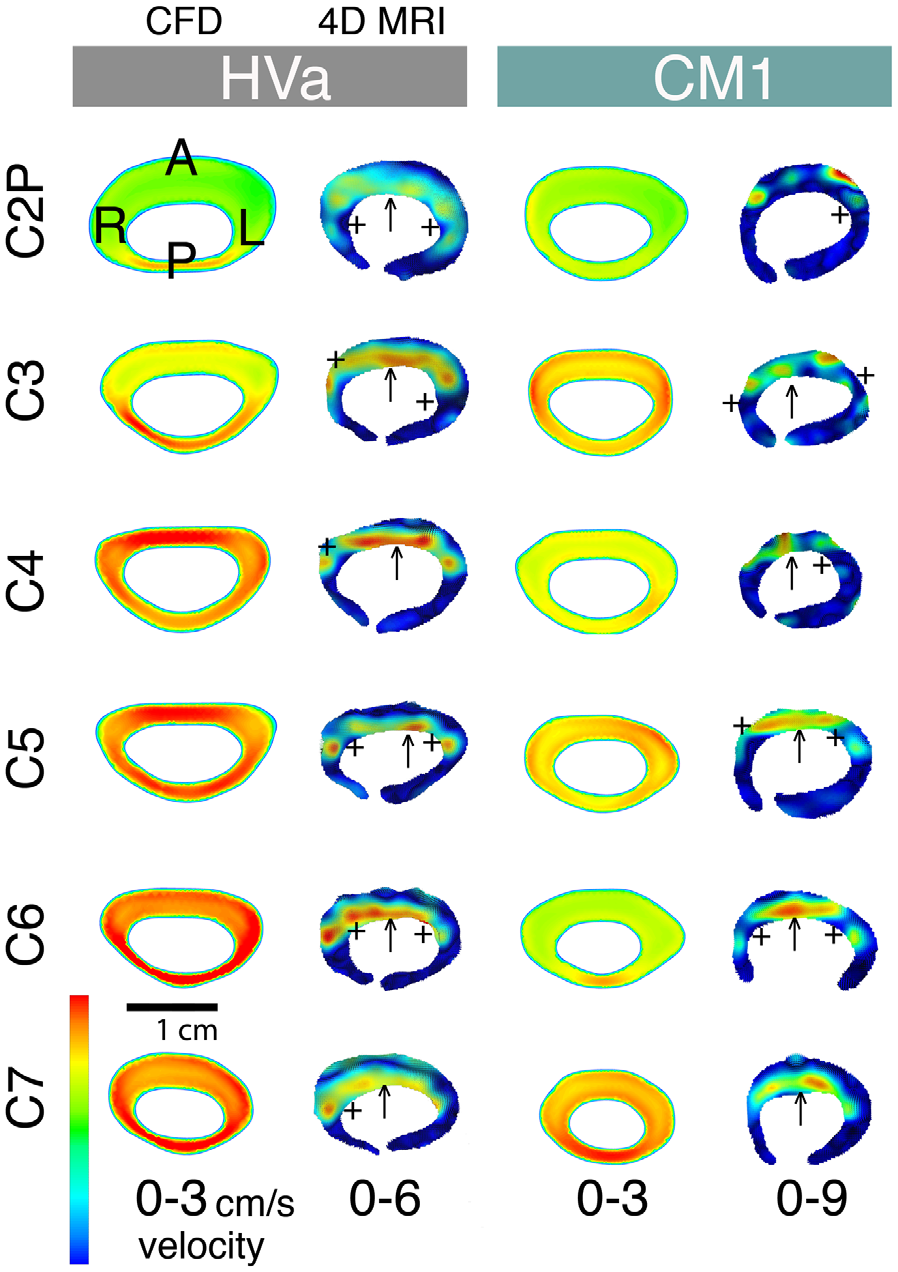
Peak-systolic thru-plane CSF velocity profiles for a healthy subject and a CM patient. Comparison of the peak systolic thru-plane CSF velocity profiles between the 4D PC MRI and CFD for HVa (left) and CM1 (right). Note the different velocity scales for each plot (optimized for visualization of flow profiles in each case). Colors indicate the magnitude of thru-plane velocities. ↑ symbols highlight the elevated anterior CSF velocities in comparison to the posterior that were observed in all of the 4D PC MRI velocity profiles (healthy and patients). The posterior versus anterior flow differences were not present in the CFD simulations (see Figure 5). +symbols indicate locations where the nerve roots appear to local CSF velocities.

Visual inspection of the 4D PC MRI and CFD thru-plane velocity profiles at peak systole revealed large spatial differences in flow patterns (Figures 5 and 6). Colours indicate the magnitude of thru-plane axial velocity (caudal direction). Greater CSF velocities were observed by 4D PC MRI in the anterior SSS in comparison to the posterior space in all healthy subjects and CM patients. In contrast, relatively uniform CSF flow profiles were simulated by CFD. Two of the four Chiari patients (CM 3 and 4) showed flow jets on the 4D PC MR images (see CM3 at FM and C1; CM4 at FM, C1 and C2M). No such flow jets were present in the corresponding CFD velocity profiles. The flow jets were unilateral in both subjects. Velocity profile was skewed to the narrower posterior subarachnoid space in a number of the CFD simulations (see HVa at C3; HVb at C2P, C3 and C4; HVc at C3 and C6; CM2 at C7; CM3 at C2P, C6 and C7; CM4 at C2P). In the 4D PC MRI images, velocity profiles were not skewed to the posterior subarachnoid space in any of the measurement planes. Instead, relatively high and concentrated regions (jets) of CSF flow were observed throughout the anterior subarachnoid space for the healthy and CMI group 4D PC MRI measurements.

Figure 6 shows a detailed view of the 4D PC MRI and CFD velocity profiles for a healthy subject (healthy a) and CM1 patient (from C2P – C7 level). The ↑ and + symbols highlight anterior dominated CSF flow and reduced CSF velocities near nerve roots, respectively. In HVa at C2P and C3, and in CM1 at C6, the CFD velocity profiles are skewed posterior to the cord while in all of the 4D PC MRI planes the velocity profile is skewed to the anterior to a great degree. The velocity profile at peak systole measured by 4D PC MRI was much rougher than the smooth uniform velocity profiles simulated in CFD. Localized velocity jets were observed on each side of the cord in HVa and to a lesser degree in CM1.

### Motion of the Cerebellar tonsils

**Figure 7:**
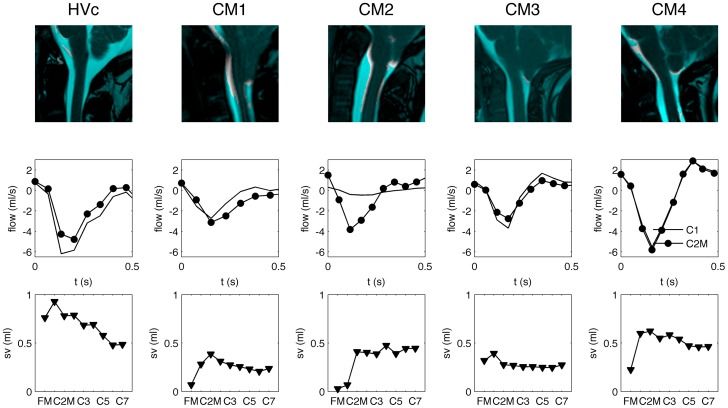
Motion analysis of the MRI images for healthy subject c (Hty c) and CM patients. Pixels in the image that are not masked in blue indicate tissue regions of the brain/spinal cord that move during the cardiac cycle. The larger the region, the greater the tissue motion; e.g. CM1, CM2 and CM4 appear to have the greater level of tissue motion in comparison to CM3. Unsteady CSF flow measured at the C1 and C2M is shown in the center row for each patient. CSF stroke volume (SV) at each axial location along the SSS (FM – C7) is shown in the bottom row for each subject.

Motion of the cerebellar tonsils during the cardiac cycle is depicted in Figure 7 (top row) for the four CM patients and one healthy subject (hvc). Regions without a blue mask colour highlight tonsillar motion. As a whole, healthy subjects had less tonsillar motion than the CM patients. CM 1, 2 and 4 had greater tonsillar motion than CM 3. Motion of the spinal cord was also noted near the brain stem in CM1 and CM2, while in CM3 and CM4 little motion was present at the brain stem.

CSF flow over the cardiac cycle at C1 and C2M vertebrae level (middle row) and total SV at various axial locations along the SSS (bottom row), as obtained from the 4D PC MRI measurements, are shown below the tonsillar motion image for each subject. The CSF flow waveform at C1 and C2M was very similar in HVc, CM3 and CM4. In CM1 and CM2 the waveform varied a great degree in terms of shape and amplitude.

Stroke volume (SV) varied a great degree at different axial locations along the spine for the subjects in our study. At the FM, SV was greatest in healthy subjects at about 0.76 ml per CSF flow cycle. In the CMI patients, SV at the FM varied from nearly zero, in CM1 and CM2, to approximately 0.3 ml, in CM3 and CM4. Interestingly, the two patients with the greatest reduction in SV (CM1 and CM2) at the FM had the greatest brain motion. The two patients with a smaller level of brain motion had a smaller reduction in SV near the FM. Below the C2P level, SV decreased along the spine in the healthy subject while in the CM patients SV remained fairly uniform.

### Independence Studies

**Figure 8:**
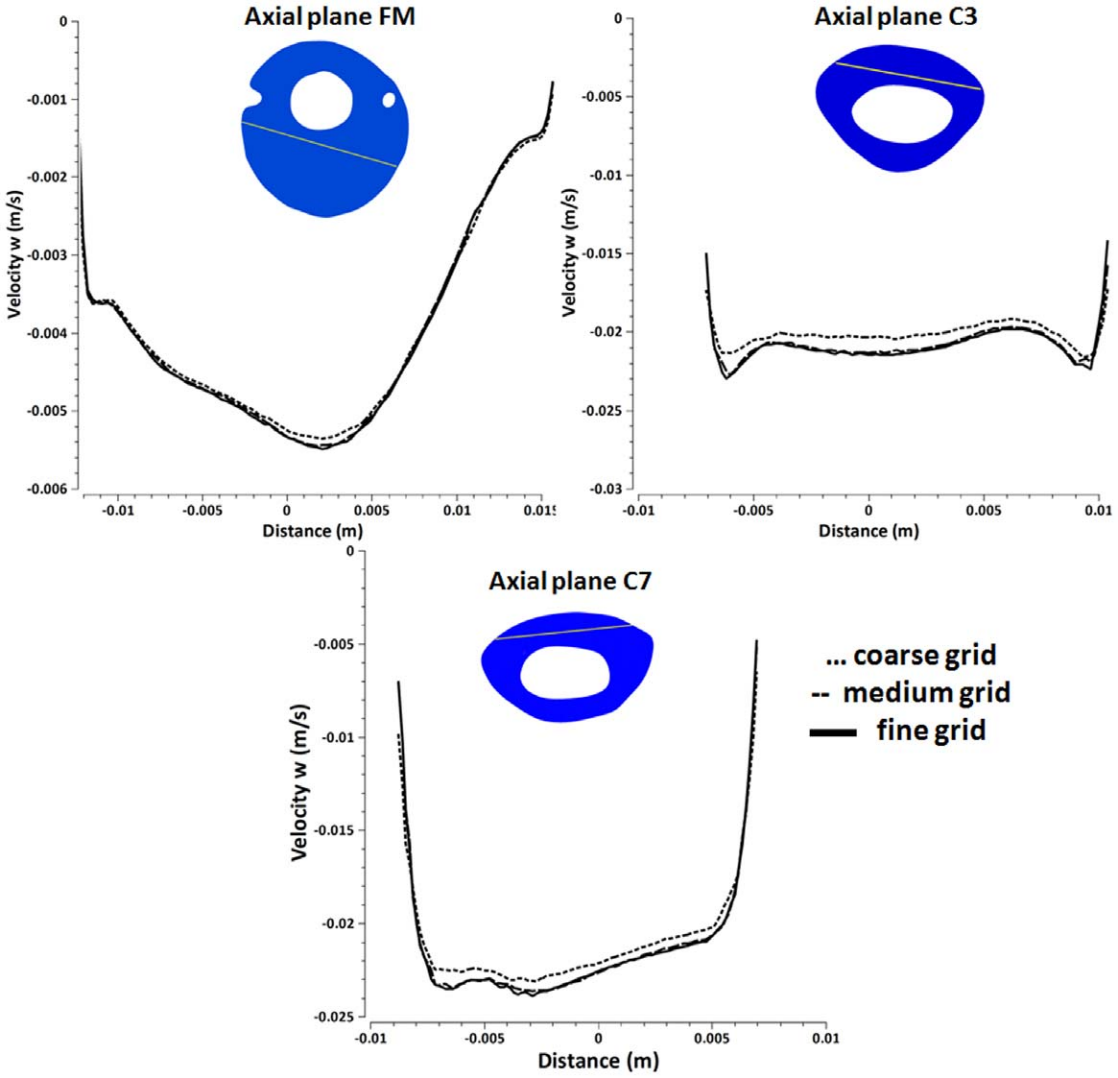
Velocity-w at axial locations through the spinal cord for three grids of different density. Plots of peak systolic velocity in the z direction (velocity w) along vectors through the cervical spinal cord for three different axial locations as calculated with three grids (a) Velocity-w along the vector at the cross-section of axial plane FM, (b) Velocity-w along the vector at the cross-section of axial plane C3 (c) Velocity-w along the vector at the cross-section of axial plane C7.

Figure 8 shows the z-direction velocity, Velocity w at peak systole for a selected vector within the axial planes located at FM (a), C3 (b), C7 (c) for the coarse, medium and fine grid simulation performed with a *T*/1,000 time step size. Time-step independence studies showed graphically indistinguishable results, especially in the cases of the medium and fine mesh. The maximum relative error, *e*, was 20% for the coarse to medium grid and 5% for the medium to fine grid.

## Discussion

In the present study we focus on analysis of CSF dynamics present in the cervical SSS by comparing 4D PC MRI measurements to subject-specific rigid wall and anatomically simplified CFD simulations. Our goal was to compare these two possibly important techniques to better understand their potential to assess CSF dynamics in healthy and diseased conditions. We compared the two techniques in terms of 1) peak velocities and 2) visual inspection of velocity profiles since both of them are regarded as possible indicators of symptomatic patients with CM. As such, those two factors, when visualized with 4D PC flow imaging and in combination with CFD simulations, may help to more precisely identify patients who are likely to benefit from craniocervical decompression.

Our results showed that the 4D PC MRI measurements and CFD simulations did not have similar CSF dynamics in terms of peak velocities or velocity profiles over a heterogeneous range of CSF flow conditions in terms of age, sex and pathology. These differences were more pronounced in CM patients particularly near the FM. We hypothesize that the differences can be accounted for due to i) neglect of small structures and/or tissue motion in the cervical SSS in the CFD simulation and ii) noise in the 4D PC MRI measurements.

### Potential Reasons for Different Peak CSF Velocities

Our results supported that thru-plane peak systolic velocities were consistently greater in magnitude for the 4D PC MRI measurements than the CFD simulations in both healthy subjects and CM patients (Figure 4). These velocity differences were more pronounced in CM patients compared to healthy volunteers, in particular at the level of the FM. The lower peak velocities in the CFD simulations in comparison to the 4D PC MRI measurements could be due to the following reasons a) overestimation of the SSS cross-sectional area, b) underestimation of the CFD input flow boundary condition, c) non-uniform porosity of the SSS, d) structural motion of the neural tissue, e) noise in the 4D PC MRI measurements. We conjecture c, d, and e to be the most plausible.

#### a) Overestimation of the SSS cross-sectional area

The 3D CFD geometry was manually segmented based on the high-resolution VISTA MRI geometry scans. It is possible that the CFD geometry cross-sectional area was larger than *in vivo* thus resulting in lower peak velocities due to the linear relation of velocity and cross-sectional area for an incompressible fluid moving in a rigid conduit. However, we do not expect this to be the case since the velocity differences occurred along the entire cervical spine and because these differences were 4X greater in some cases. For this to occur, the manual segmentation would need to be incorrect by a factor of four or more. Nonetheless, in our study the manual segmentation for each subject was checked by two radiologists and confirmed to be representative of *in vivo*.

#### b) Underestimation of the CFD input flow boundary condition

Similar to overestimation of cross-sectional area, underestimation of the input flow boundary condition for the CFD simulation would result in lower peak velocities. However, our methods involved carefully specifying the input boundary condition with the greatest peak flow rate and thus we do not expect an underestimation of the flow boundary condition but rather possibly an overestimation. The CSF flow waveform amplitude, that was quantified at different axial levels by 4D PC MRI, was found to vary along the cervical spine; presumably due to compliance of the SSS (see Figures 5, 6 and 7) [11]. To some extent, these variations may be attributed to noise in the 4D PC MRI signal in regions with low velocities and structural motion of the tissue (see below for more on structural motion). For the CFD simulation flow boundary condition, we chose to use the CSF flow waveform with the greatest peak flow value (caudal direction) reasoning that at this location signal to noise would be better than other regions. The selected location was at C1, C2M or C3 for the study population.

#### c) Inhomogeneous porosity of the SSS

Our 4D PC MRI measurements show a dominance of anterior CSF velocities in comparison to the posterior cervical SSS (Figures 4 and 5). In contrast, the CFD simulations did not show anterior dominance of CSF velocity in any of the simulations. Instead, the velocity profile was skewed to the narrower posterior subarachnoid space in a number of the CFD simulations. We suspect that these differences are due to inhomogeneous distribution of arachnoid trabeculae or other fine anatomical structures that result in preferential CSF movement through the anterior SSS in the cervical SSS. However, the CFD simulation considered the SSS to be a fluid continuum in which the fine anatomical structures were neglected. If these structures were present the SSS cross-sectional area would be reduced and thus peak velocities would increase. Additionally, a study in the literature has shown that the arachnoid trabeculae were more densely packed in the posterior SSS [38]. Under this condition CSF would move more freely on the anterior SSS and thus CSF velocities in this region would be greater.

#### d) Structural motion of the neural tissue

The CFD simulation did not take into account structural motion of the neural tissue. However, it was clear that structural motion was present, particularly in the patients near the FM (Figure 7). Unsurprisingly, the CFD and 4D PC MRI results deviated from one another to the greatest degree in patients near the FM. The motion analysis of the cerebellar tonsils during the cardiac cycle showed descent of the tonsils during systole. Thus, at this time point the cross-sectional area of the SSS would be reduced and make CSF velocities in this region increase. However, the motion of the tonsils was not taken into account by the CFD simulation likely resulting in lower peak velocities. While tissue motion at the cerebellar tonsils may account for velocity differences near the FM, it would not account for velocity differences in the middle/lower cervical spine that were observed in our study where little tissue motion was observed in any of the subjects. As a result, in these regions it is more plausible that peak flow differences were due to either a, b, or c as mentioned above.

#### e) Noise in the 4D PC MRI measurements

It has been argued above that the difference in 4D PC MRI and CFD results can be accounted for by oversimplification of the CFD simulation. However, it should be noted that the 4D PC MRI measurement methodology also needs improvement. Phase contrast imaging requires a maximum measurable velocity to be set so as to balance noise and phase aliasing. In order to correctly detect high velocities and avoid aliasing artifacts, the sequence presets had to be adjusted to higher velocity encoding factors in Chiari patients than healthy volunteers (see methods). By choosing a higher velocity encoding factor, the sensitivity for the detection of slow flow components was reduced and may have led to an underestimation of slow flow. By these means, overall flow rates which were used as inlet flow boundary conditions may have been underestimated. New techniques using multiple velocity encoding schemes aim at increasing the overall sensitivity for a wider range of flow velocities and reduce the velocity-to-noise ratios [39].

Bunck et al. [40] evaluated the accuracy of the 4D PC MR sequence by comparing CSF flow velocities as measured by a conventional 2D PC MRI to 4D PC MRI sequence at four representative sites of the cervical canal. The comparison showed an overall good agreement of peak velocities in healthy volunteers with only a small bias. With no 2D PC data acquired in their patient population, future studies are required to assess how 4D PC MRI could be compared with conventional 2D PC flow imaging and whether it adds clinically valuable information. Long acquisition times make the 4D PC imaging prone to motion artifacts that could increase the noise level. This technique also requires a significant level of pre-processing and filtering of the data for analysis. Each step of post-processing can introduce error to the measurements.

### Different Velocity Profiles and Importance of Small Anatomy

The 4D PC MRI CSF velocity profiles showed a strong dominance of flow on the anterior SSS in comparison to the posterior (Figures 5 and 6) while CFD velocity profiles were fairly uniform along the cervical spine except near the FM in CM 1 and CM 2 patients. One might argue that the 4D PC MRI measurements are suspect since they have spatial and temporal limitations. However, the MRI flow measurement generally improves with flow velocity. Thus, while the noise that is present on the posterior spinal cord 4D PC MRI measurements does make the exact flow profile in this region suspect, it does not mean that an overall dominance of CSF flow would not be noticed. In the present case velocities were relatively high anterior to the SC and thus one would still be capable of delineating flow dominance on one side of the SC or another.

The differences in velocity profiles and peak velocities between the 4D PC MRI measurements and CFD simulations suggest that the level of anatomical detail in CFD simulations are not adequate to accurately model the CSF dynamics in the cervical spine. The differences in anterior versus posterior flow in the 4D PC MRI measurements appear to be important in the overall flow field. However, the CFD did not capture the level of anterior flow dominance. Thus, SC nerve roots, denticulate ligaments and/or other small anatomical structures such as the arachnoid trabeculae may be required to accurately model the flow field. It is yet clear if all or just some of these anatomical structures need to be included.

Various researchers have completed computational studies including different aspects of small structures in the SSS (see Table 1). Nevertheless, none of these studies have included all of the anatomical fine structures in their computational model, including the subject-specific geometries and flow boundary conditions and compared their simulation results with 4D PC MRI or 2D phase-contrast MRI measurements. Neglecting anatomical details makes the CFD simulations simpler and require less computing time [2]; however it may not be representative of the *in vivo* flow field.

While the present study did not include the small structures in the CFD simulations, it did compare directly the CFD results with the *in vivo* 4D MR measurements in healthy subjects and CM patients. One reason for the lack of comparison in the literature is that the 2D phase contrast MRI images are generally obtained with a slice thickness greater than the nerve root dimensions thus washing out some of the spatial flow complexity. Therefore, the single direction of velocity encoding does not permit quantification of the more complex flow phenomena that might arise near fine structures. Additionally, fine structures within the SSS are difficult to be captured with the current imaging techniques. Sigmund et al. [41] recently utilized 7T MRI with a custom designed neck coil to obtain high–resolution anatomical images of the cervical SSS with as low as 180 micron isotropic resolution. This resolution has potential to geometrically define nerve roots and denticulate ligaments but not arachnoid trabeculae.

### Importance of Tissue Motion

Tissue motion appeared to relate with CSF dynamics near the FM. It appeared that differences between C1 and C2M level CSF flows and stroke volumes could be related to tissue motion of the brain (Figure 7). In particular, greater changes in CSF stroke volume were present near the FM in subjects with greater brain tissue motion. It can be hypothesized that abnormally elevated brain tissue motion in CM patients could result in movement of CSF by displacement. However, more patients and healthy subjects would need to be analysed to validate this hypothesis. Cousins et al. [42] measured tonsillar motion with CINE MR imaging in patients suspected to have CM and subjects without any tonsillar ectopia. They found that patients and subjects with normal cerebellar tonsils both depicted a small-amplitude tonsil movement in cephalad and caudal directions during the cardiac cycle.

### Limitations

There were a number of limitations in this study in terms of: 1) study population, 2) 4D PC MRI flow imaging methods and 3) CFD methodology. The primary aim of the study was to compare quantification of CSF dynamics by 4D PC MRI and CFD under a variety of CSF flow situations. Thus, a limited study population was selected to encompass both healthy subjects and CM patients that depicted a variety of CSF flow patterns. We chose four CM patients with differences in flow alterations, severity of tonsillar herniation and symptoms. The healthy subjects were considerably older than the CM patients and thus were also likely to have different flow characteristics [9], [43]. In addition, several factors were not controlled including neck angulation that might have had an impact on CSF dynamics [44], [45], [46], [47]. Future studies should be performed in a larger population with age-matched controls. It would also be useful to conduct repeatability studies.

The 4D PC MRI methods presented a number of important limitations. Slow moving CSF velocities were difficult to obtain due to inherent lack of signal and/or noise in the 4D PC MRI and relatively high velocity encoding values needed. This was particularly in the case of CM patients where flow jets were present within the ROI near FM and C1 level. The 4D PC MRI post-processing tool had limited ability for ROI selection and made it difficult to define complex geometries such as near the FM. At the FM avoidance of high arterial blood flow velocities from the vertebral arteries was difficult and altered the ROI. Future improvements in the 4D PC MRI post processing could be achieved by a more robust pixel selection technique such as a point-by-point selection that incorporates spectral analysis and/or cross-correlation of pixel velocities.

To define the geometric region used for the CFD simulation we utilized an MRI scan with a spatial resolution of approximately 1 mm. This scan provides limited details about the fine anatomy that appeared to be an important factor in our study. It would be helpful to utilize images of higher resolution to define the geometric boundaries such as those that can be obtained with 7T MRI. Flow boundary conditions for the CFD model were difficult to define due to differences in CSF flow amplitude. A more accurate CFD simulation of the cervical CSF might incorporate the fluid structure interaction of the spinal cord, dura and other structures. It may also be required to incorporate moving boundary methods to model the tonsil and/or spinal cord motion in CM patients. Similar to previous studies in the literature, we set the pressure boundary condition to zero at the flow outlet. However, at this region there was at times bifurcating and/or complex flow outlet geometry. It is expected that the *in vivo* pressure could be different for the outlets and thus would impact CSF flow velocities. Even with these alterations in flow, we do not expect them to propagate further down the spine where the pressure around the spinal cord would likely be relatively uniform.

### Conclusion

This study represents the first comparison of 4D PC MRI measurements and CFD simulation of CSF motion in the cervical SSS for healthy subjects and CM patients. CSF dynamics were found to be considerably different in 4D PC MRI versus CFD simulations. We believe the deviation of CFD results from the 4D PC MRI measurements is likely due to neglect of small anatomical structures in the cervical SSS and tissue movement. Thus, the present anatomically simplified rigid wall CFD methods likely need to be improved to accurately model CSF dynamics in the cervical SSS in terms of peak flow velocities and velocity profiles. Further analysis, such as incorporation of the spinal cord nerve roots and/or denticulate ligaments and an *in vitro* study, should be conducted to understand the differences in flow fields between the two methods. The results of our study also highlight the utility of CFD in conjunction with 4D PC MRI for detailed analysis of CSF flow dynamics that could help distinguish physiological from complex pathological flow patterns at the FM and cervical SSS. However, a full understanding of why pulsatile motion of the CSF is needed to maintain craniospinal health remains enigmatic. We expect that a combination of 4D PC MRI measurements and CFD simulations will be key tools to help assess and understand the CSF dynamics in health and disease states.
